# Mitogen Synergy: An Emerging Route to Boosting Human Beta Cell Proliferation

**DOI:** 10.3389/fcell.2021.734597

**Published:** 2022-01-27

**Authors:** Ekaterina Shcheglova, Katarzyna Blaszczyk, Malgorzata Borowiak

**Affiliations:** ^1^Institute of Molecular Biology and Biotechnology, Faculty of Biology, Adam Mickiewicz University, Poznań, Poland; ^2^Department of Molecular and Cellular Biology, Baylor College of Medicine, Houston, TX, United States

**Keywords:** endocrine beta cell, proliferation, diabetes, synergy, signaling, human vs. rodent

## Abstract

Decreased number and function of beta cells are a key aspect of diabetes mellitus (diabetes), a disease that remains an onerous global health problem. Means of restoring beta cell mass are urgently being sought as a potential cure for diabetes. Several strategies, such as *de novo* beta cell derivation via pluripotent stem cell differentiation or mature somatic cell transdifferentiation, have yielded promising results. Beta cell expansion is another promising strategy, rendered challenging by the very low proliferative capacity of beta cells. Many effective mitogens have been identified in rodents, but the vast majority do not have similar mitogenic effects in human beta cells. Extensive research has led to the identification of several human beta cell mitogens, but their efficacy and specificity remain insufficient. An approach based on the simultaneous application of several mitogens has recently emerged and can yield human beta cell proliferation rates of up to 8%. Here, we discuss recent advances in restoration of the beta cell population, focusing on mitogen synergy, and the contribution of RNA-sequencing (RNA-seq) to accelerating the elucidation of signaling pathways in proliferating beta cells and the discovery of novel mitogens. Together, these approaches have taken beta cell research up a level, bringing us closer to a cure for diabetes.

## Introduction

### Diabetes and the Replication of Beta Cells

The beta cells, together with alpha, delta, PP cells and rare epsilon cells, compose the islets of the endocrine pancreas. Beta cells are highly specialized in the production and secretion of insulin, a hormone enabling glucose transfer from the blood to cells, for direct use or storage as an energy source (Guney et al., 2020). An impairment of glucose homeostasis may result in hyperglycemia and diabetes mellitus (commonly termed diabetes). This metabolic disease is one of the most onerous health problems worldwide, with more than 460 million people (∼9% of the total world population) affected, and about 10% of all-cause mortality attributable to diabetes. Moreover, diabetes is a growing problem, with the number of cases annually tripling over the last 20 years (from 151 million in 2000 to 463 million in 2019), and predictions that there will be 700 million cases by 2045 (Saeedi et al., 2019). The two major types of diabetes differ in terms of their etiology and severity. Type 1 diabetes (T1D) occurs due to the autoimmune destruction of beta cells (Katsarou et al., 2017), whereas type 2 diabetes (T2D) has a more complex mechanism, involving insulin resistance in peripheral tissues (Zaccardi et al., 2016). However, a crucial aspect of both types of diabetes is the presence of an insufficient number of functional beta cells. Efforts are, therefore, being made to develop effective treatments or cures based on the restoration of beta cell functional mass and, thus, glucose homeostasis.

In healthy humans, beta cells account for about 50% of the islet cells, on average (Brissova et al., 2015; Roscioni et al., 2016) and the total beta cell mass is about 1–2 g (Wang et al., 2015d). Beta cells are long-lived, slowly replicating cells (Cnop et al., 2010, 2011). Most human beta cells form during embryogenesis. About 2–3% of beta cells replicate during infancy and early childhood (Meier et al., 2008; Gregg et al., 2012; Wang et al., 2015d), but the capacity of these cells subsequently declines. The beta cell population is fully established by the start of adulthood, and the beta cells remain practically quiescent throughout the rest of the individual’s life (Cnop et al., 2011), with rare exceptions, as discussed below. The estimated replication rate of adult beta cells is 0.1–0.5% (Meier et al., 2008; Gregg et al., 2012; Wang et al., 2015d). Under prolonged increases in insulin demand, these rare proliferation events may not be sufficient to replace the destroyed beta cell mass, leading to diabetes. Given the increasing incidence of diabetes, there is now an urgent need for robust approaches to restoring beta cell mass.

### Strategies for Replenishing Beta Cell Mass

Pancreatic islet transplantation from donors is a recognized approach for replacing lost or damaged beta cells. In 2000, the landmark Edmonton protocol successfully demonstrated islet transplantation from cadaveric donors and consequently, a resolution in hyperglycemia (Shapiro et al., 2000, 2006; Ryan et al., 2001) and exogenic insulin independence. Islet isolation and transplantation techniques have since improved, resulting in a need for fewer donors and a lower risk of surgery-associated complications (Bottino et al., 2018). However, the shortage of cadaveric donor islets and the need for life-long immunosuppression limit this approach to a very small group of patients (Bruni et al., 2014; Helman and Melton, 2021). Efforts are therefore currently ongoing to identify alternative sources of human beta cells.

One of the most promising ideas for a robust source of new beta cells involves the differentiation of human pluripotent stem cells (hPSCs). With their self-renewal and ability to differentiate into any cell type, hPSCs can be used to generate large numbers of human beta cells. Current hPSC differentiation protocols yield human beta-like cells in 20–30 days, with a yield of 40–50% successfully differentiated beta cells at the final stage. However, not all *in vitro*-derived beta cells have the physiological insulin-secretion characteristics of primary beta cells (D’Amour et al., 2006; Kroon et al., 2008; Tateishi et al., 2008; Zhang et al., 2009; Zorn and Wells, 2009; Nostro et al., 2011; Rezania et al., 2012, 2014; Hrvatin et al., 2014; Pagliuca et al., 2014; Russ et al., 2015; Nair et al., 2019; Rosado-Olivieri et al., 2019; Velazco-Cruz et al., 2019; Veres et al., 2019). Nevertheless, hPSC-derived cells restore euglycemia in diabetic mice, and it remains to be established whether they are also capable of restoring glucose homeostasis in humans (reviewed in Helman and Melton, 2021).

As an alternative, transdifferentiation can be used to convert mature somatic cells directly into beta cells. During transdifferentiation the profile of the cell gradually changes from the non-beta to beta cell with the intermediate polyhormonal profiles. It is not well understood whether the transdifferentiation cells become a kind of “chimera/hybrid” cell that retains some of its original features. Yet, it might be critical to evaluate it in detail as for example different types of endocrine cells within islets regulate each other’s physiology.

Zhou et al. (2008) were the first to demonstrate the successful redifferentiation of pancreatic exocrine cells in adult mice into cells closely resembling beta cells, through the ectopic expression of three key beta cell regulatory transcription factors — MafA, Pdx1, and Ngn3 — in acinar cells. *In vitro* transdifferentiation into beta cells has also been achieved with murine non-pancreatic cells, including endocrine cells in the intestine or stomach (Chen et al., 2014; Ariyachet et al., 2016; Baeyens et al., 2018). In mice with nearly complete loss of beta cells, the other endocrine cells like alpha and delta cells can transdifferentiate into insulin producing cells (Thorel et al., 2010; Chera et al., 2014; Chakravarthy et al., 2017).

Moreover, human alpha cells reprogrammed *in vitro* into beta cells and engrafted into diabetic mice have been shown to produce insulin and restore glucose homeostasis (Furuyama et al., 2019). However, there are currently substantial obstacles to the use of transdifferentiation for clinical applications. For example, it remains unclear how similar the transdifferentiated beta cells are to native beta cells and whether they display reconversion over time. Furthermore, transdifferentiation is currently conducted predominantly with viral vectors, the safety of which for human applications has yet to be demonstrated (van der Meulen and Huising, 2015; Osipovich and Magnuson, 2018; Nair et al., 2020). Some clinically approved drugs, like neurotransmitter GABA (Ben-Othman et al., 2017) and the anti-malarial drug Artemisinin (Li et al., 2017) have been reported to induce alpha- to beta cell conversion. However, those findings have failed to be reproduced by subsequent independent studies (Ackermann et al., 2018; van der Meulen et al., 2018; Shin et al., 2019), leaving the potency of these drugs to induce alpha-to-beta cell conversion questionable.

The third strategy for the *de novo* generation of human beta cells is based on the identification of progenitors or stem cells in the adult pancreas. It remains a matter of debate whether these cells are present in significant numbers in the adult mouse or human pancreas, and under what physiological conditions (Domínguez-Bendala et al., 2019).

The hypothesis that pancreatic stem or progenitor cells might exist in the adult pancreas (Bonner-Weir, 2000) was initially based on histological observations of single islet cells or small islets embedded in or closely associated with adult rodent and human pancreatic ducts, suggesting the emergence of new islet cells from ducts. However, this view was challenged in 2004, when, using a lineage-tracing (LT) methodology, Dor et al. (2004) did not find any evidence of beta cell neogenesis in the pancreas of adult mice. Instead, the results of the LT-labeling showed that the new beta cells formed during the period from 2 to 12 months in adult mice originate from the already existing beta cells. Coherently with this finding, Kushner laboratory showed that new beta cells in adult mice were generated by the replication of existing beta cells rather than by the differentiation of progenitor or stem cells (Teta et al., 2007) using DNA-analog based lineage tracing technique to mark multiple rounds of beta cell proliferation *in vivo*.

Interestingly, Wang et al. (2020) recently identified Procr^+^ cells in the adult pancreas by single-cell RNA sequencing (scRNA-seq). These cells may be adult stem cells, as Procr is a surface marker of adult stem cells in other mouse tissues (Wang et al., 2015a; Yu et al., 2016; Fares et al., 2017). However, the potential of these cells to act as adult pancreatic stem cells, and their differentiation potential and physiological role remain unclear.

Diabetes is often associated with beta cell dedifferentiation therefore, another promising approach is to identify signals that can prevent or reverse the beta cell dedifferentiation. Using single cell RNA-seq and streptozotocin-induced diabetes in mice (Sachs et al., 2020) recently showed that insulin treatment restores beta cell function. Furthermore, beta cell specific delivery of estrogen as conjugate with glucagon-like peptide-1 decreases insulin requirements by 60%, triggers activation of the endoplasmic-reticulum-associated protein degradation system, and further increases beta cell survival and regeneration. This study reveals the potential of pharmacological priming of dedifferentiated beta cells for diabetes remission.

A detailed discussion of the abovementioned strategies for increasing the beta cell population is beyond the scope of this review and can be found elsewhere (Basile et al., 2019; Nasteska et al., 2019; Tan et al., 2019; Guney et al., 2020; Helman and Melton, 2021). Here, we summarize and discuss recent advances in the field of human beta cell proliferation, focusing more closely on the contributions of mitogen synergy and transcriptomics to elucidating the signaling pathways and genes involved in beta cell proliferation, providing hints for the discovery of novel mitogens.

## Beta Cell Proliferation

### When Do Adult Beta Cells Proliferate?

Research on beta cell proliferation has suffered from both inconsistency and controversy. Adult beta cells were initially considered to be post-mitotic, due to the technical impossibility of detecting their rare replication events at the time (Richardson and Young, 1938). Developing and immature beta cells show higher proliferation potential. For example, the EndoC-βH1 cell line derived from human fetal beta cells (Ravassard et al., 2011) responds to mitogens differently than adult beta cells. EndoC-βH1 cells reach 20% EdU labeling index upon CHIR99021 treatment (Tsonkova et al., 2018). CHIR99021, a GSK-3 beta inhibitor, in adult human dispersed beta cells did not show mitogenic effects when applied alone, although in combination with harmine it increases Ki67 labeling from 0 to 3.5% (Ackeifi et al., 2020a). These differences may be partially explained by signals from the microenvironment of the developing pancreas, principally mediated by the extracellular matrix (ECM). Defining those cues and the perspective of their tailored employment in cell reprogramming toward beta cells is one more intriguing branch of research. Research conducted over the past several years has revealed many differences between the mechanisms by which embryonic and post-weaning beta cell mass are expanded and maintained. Many of the genes important in embryonic beta cell development have distinct roles in maintaining adult beta cell identity and functionality, e.g., transcription factor MafB is critical to the development of mature beta cells but postnatally expressed in alpha cells (Artner et al., 2006, 2007) or connective tissue growth factor CTGF-required for beta cell proliferation during development but not in adulthood (Gunasekaran et al., 2012). In addition, specific cell cycle regulators (CCRs) also differ in their function between embryonic and adult beta cells. Expression of p16Ink4a, a cell cycle inhibitor, increases in beta cells with age and contributes to the age-related decline in beta cell proliferation (Krishnamurthy et al., 2006). As fetal beta cells are generally considered immature, the higher replication rate of beta cells is linked to immaturity defined as physiological incompetence.

However, many reports over the years have demonstrated that adult beta cells do have the capacity to replicate, but that this capacity is limited (Chick and Like, 1971; Granger and Kushner, 2009). It is currently widely accepted that the beta cell mass of adults can increase significantly during states of intense metabolic demand, such as pregnancy, obesity, or injury (e.g., partial pancreatectomy) (Rieck and Kaestner, 2010). Compensatory beta cell growth is well documented in rodents (Parsons, 1992; Granger and Kushner, 2009; Bonner-Weir et al., 2010; Keenan et al., 2010; Linnemann et al., 2014). However, in humans, obesity (Saisho et al., 2013) and pregnancy (Van Assche et al., 1978; Butler et al., 2010) have a smaller impact on beta cell replication, and pancreatectomy has been reported not to induce human beta cell proliferation (Menge et al., 2008). The increase in beta cell mass was demonstrated to occur due to the replication of existing beta cells rather than neogenesis (Granger and Kushner, 2009; Rieck and Kaestner, 2010). Some studies have also suggested that beta cell neogenesis may occur during pregnancy in humans, but the mechanisms involved remain unclear (Butler et al., 2010; Gunasekaran and Gannon, 2011; Basile et al., 2019). Importantly, these findings imply that beta cells display a certain degree of plasticity in the regulation of their mass (Roscioni et al., 2016). Since in both types of diabetes, some beta cells with residual functionality may be retained in the pancreas (Pipeleers et al., 2008; Rahier et al., 2008; Kahn et al., 2009; Keenan et al., 2010; Oram et al., 2014), these cells could potentially serve as a source for restoration of the beta cell population. Approaches aiming to restore glucose homeostasis via beta cell replication may be particularly suitable for T2D patients, due to the larger pool of residual beta cells and the absence of autoimmune beta cell destruction in these patients. Expansion of the existing beta cell population via replication may also be considered a more “natural” or direct way of obtaining more beta cells. However, human beta cells are not prone to replicate, and there is therefore a need to identify stimuli which enable them to increase replication. Further, the difficulty in induction of human beta cell replication is intensified by the growing evidence that rodent models do not accurately recapitulate characteristics of human beta cell replication (Wang et al., 2015d) and consequently findings from rodent models often do not translate to human beta cells.

### Why Do Beta Cells Have Such Low Rates of Proliferation?

The replicative capacity of beta cells decreases with age, and the proliferation of these cells is inversely correlated with their functional maturation. Human adult beta cells have a proliferation rate below 0.5%, which may appear low at first sight, but is nevertheless sufficient to maintain a stable beta cell mass under normal physiological conditions throughout the life of the individual, with the replenishment of dying beta cells and constraints on unnecessary proliferation. Unlike highly regenerative tissues, such as the liver or intestine, which are directly exposed to toxins and other exogenous agents (Schmucker and Sanchez, 2011; Kopp et al., 2016), beta cells perform their physiological duties in a relatively “toxin- and mechanical force-free” environment. They do not, therefore, need to have a high regenerative potential. The origins of the low proliferation capacity of beta cells are unclear, but the specialization of these cells is one plausible explanation. Beta cells are terminally differentiated and highly specialized in insulin production and secretion (Gutierrez et al., 2017), with about 45% of all the mRNA produced in beta cells encoding insulin (Morán et al., 2012). While this high degree of specialization ensures that beta cells are masters of their craft, it may also be a drawback, weakening the competence of beta cells in other aspects of cell life. If this is indeed the case, then the decline in proliferative capacity during childhood described above may result from beta cells taking on insulin synthesis and secretion as their predominant functions. Furthermore, life/death decisions in beta cells are regulated by a strong unfolded protein response (UPR). The UPR is a mechanism of defense against endoplasmic reticulum (ER) stress, which occurs when the demand for the biosynthesis of proteins, such as insulin, increases (Xin et al., 2018). Under strong and persistent ER stress, the UPR can lead to beta cell apoptosis via the activation of death effectors. Conversely, if the ER stress is resolvable, the UPR promotes beta cell survival or proliferation (Xin et al., 2018; Tatsuoka et al., 2020). A high demand for insulin synthesis may lead to ER stress and, therefore, low beta cell proliferation and/or beta cell death. Unresolvable ER stress may occur in several contexts, including genetic predisposition, chronic exposure to high glucose concentrations, and UPR dysregulation (Fonseca et al., 2011).

Aging also has a negative influence on beta cell proliferation. During the aging process, the ratio of cell cycle activators to inhibitors shifts in favor of inhibitors (De Tata, 2014; Dai et al., 2017; Ahmed, 2021). The p16 cell cycle inhibitor, the expression of which increases with aging, provides an example of this (Köhler et al., 2011; Helman et al., 2016). The expression of p16 is repressed epigenetically by Enhancer of zeste homolog 2 (Ezh2). In aging beta cells, Ezh2 expression decreases, attenuating the repression of p16 (Gunasekaran and Gannon, 2011). It has also been shown that the beta cells of mice have a refractory period, a time interval immediately following division during which division cannot recur. Aging has been shown to lead to a lengthening of this period in beta cells, potentially associated with changes in the ratio of CCRs (Salpeter et al., 2010). Elucidation of the mechanisms by which resting beta cells re-enter the active cell cycle may lead to the discovery of new mechanisms regulating the increase in beta cell mass, and facilitate the development of beta cell expansion-based therapies for diabetes.

### The Cell Cycle in Beta Cells

**FIGURE 1:**
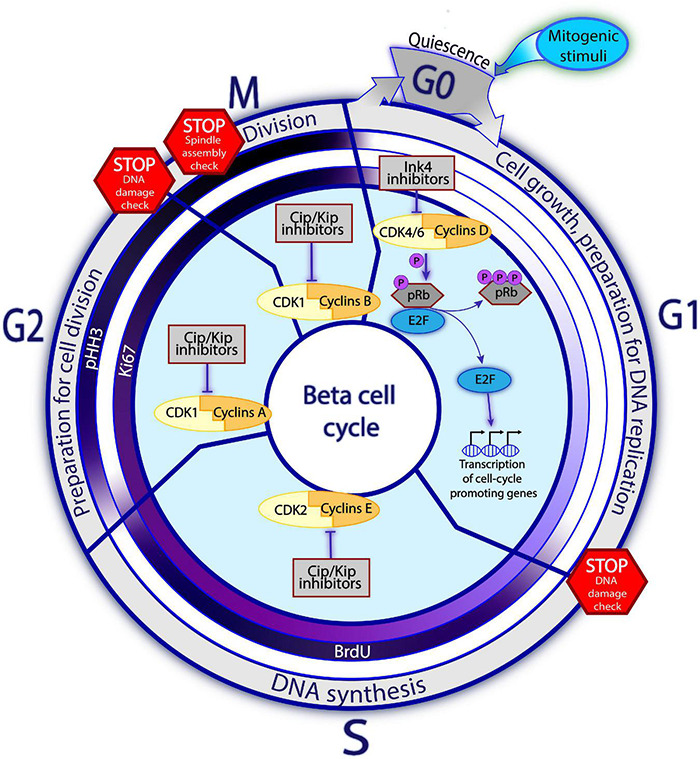
Principles of cell cycle in the beta cell. Beta cells predominantly reside in a quiescent, non-mitotic G0 phase. Upon the mitogenic stimuli, the assembly of Cyclins D and CDK4/6 complex and consequent pRb inactivation via hyperphosphorylation trigger the cell cycle entry. Once the E2F transcription factors are released from the pRb restraining, they enhance expression of the cell cycle promoting genes, including the late Cyclins and CDKs. The activities of different Cyclins-CDK complexes gradually replace each other with the cell cycle progression, so that each Cyclins-CDK complex is dominant during different cell cycle phases. In G1, Cyclins D-CDK4/6 regulate cell growth and preparation for DNA replication. In the S phase, Cyclins E-CDK2 complexes control DNA replication. Whereas in phase G2, Cyclins A with CDK1 are driving preparation for the cell division. Finally, in phase M, complexes of Cyclins B-CDK1 regulate the cell division. The promoting activities of Cyclins-CDK complexes are negatively regulated by the cell cycle inhibitors. Families of Ink4 and Cip/Kip inhibitors restrain the early and late Cyclins-CDKs complexes, respectively. On the way towards division, the cell must encounter several STOP checkpoints whereby the cell state, DNA integrity and the surrounding conditions are checked. Cells which do not pass the checkpoint, are blocked from cell cycle progression until the conditions improve or, in an irresolvable situation, undergo apoptosis. The inner circles on Figure 1 represent the commonly used proliferation markers: Ki67, BrdU, pHH3. Intensity of the violet color corresponds to the abundance of the marker at a given phase, with white color indicating absence of the marker and dark violet – the highest detectability.

Most adult beta cells are in the resting (G0) phase of the cell cycle (Hija et al., 2014). Replication is initiated when cells withdraw from G0 in response to mitogenic signals and enter the G1, S, G2, and M phases of the cell cycle (Figure 1; Frank and Tsai, 2009; Sadasivam and DeCaprio, 2013). The core of the cell cycle machinery of beta cells consists of about 30 molecules of which some act as cell cycle activators and others as cell cycle inhibitors (Fiaschi-Taesch et al., 2013b). The cell cycle activators belong to two families of proteins that can, together, form complexes: cyclins and cyclin-dependent kinases (CDKs). Cyclins follow an oscillating pattern of expression during the cell cycle (Martínez-Alonso and Malumbres, 2020). In beta cells, several cyclins, including cyclins D, E, A, B, and C (Fiaschi-Taesch et al., 2013b; Ueberberg et al., 2016), activate CDKs (Martínez-Alonso and Malumbres, 2020). These CDKs, in turn, initiate cell cycle progression through their kinase activity. CDK1, 2, 4, and 6 are present in human beta cells (Fiaschi-Taesch et al., 2013b). CDKs and cyclins are widely expressed in various cell types, but the CDK4-cyclin D1 complex seems to play a particularly important role in human beta cell replication. Cozar-Castellano et al. (2004) showed that the adenoviral delivery of CDK4 to human beta cells increased beta cell proliferation rates, which were further increased by cyclin D1 overexpression. Interestingly, despite the increase in the proliferation rate of these cells, they retained their ability to sense glucose and to secrete insulin, together with normal levels of expression for key beta cell markers (Cozar-Castellano et al., 2004).

During the G0-G1 transition, the “early” initiating complexes of CDK4/6 and cyclin D assemble and are translocated to the nucleus, where CDK4/6 hyperphosphorylates the retinoblastoma (Rb) protein, the gatekeeper of cell cycle progression. In its hypophosphorylated state, Rb restricts cell cycle progression by inhibiting transcription factors of the E2F family. Following its hyperphosphorylation, this inhibition of E2F is relaxed, enhancing the expression of genes required for cell cycle progression, including the “later” cell cycle-promoting molecules (such as cyclins E and A). Complexes of cyclin E-CDK2, cyclin A-CDK1, and cyclin B-CDK1 then assemble and promote passage into the S, G2, and M phases, respectively (Harashima et al., 2013; Salas et al., 2014; Figure 1). In human beta cells, overexpression of late complex of cyclin E and CDK1 promotes cell cycle entry. Collectively, these studies suggest that both early and late cyclins-CDKs can induce cell cycle entry, in a complementary manner, in human beta cells (Tiwari et al., 2015).

Progression through the cell cycle is controlled by cell cycle inhibitors, which by far outnumber cell cycle activators in human beta cells (Fiaschi-Taesch et al., 2013b). The suitability of internal and external conditions for cell division is checked at several checkpoints (such as the G1/S or G2/M transition points). If conditions are unfavorable, the inhibitors prevent cell cycle transition (Lim and Kaldis, 2013; Otto and Sicinski, 2017). Retinoblastoma proteins make up the major family of cell cycle inhibitors. These proteins — pRb, p107, and p130 — prevent the G1–S transition by regulating E2F-responsive genes (Harb et al., 2009; Henley and Dick, 2012). The INK4 family, including p15, p16, p18, and p19, specifically inhibits CDK4 and CDK6 activity (Quereda et al., 2016), whereas the KIP/CIP/WAF family, consisting of p21, p27, and p57, inhibits other cyclin-CDK complexes (Quereda et al., 2016; Martínez-Alonso and Malumbres, 2020; Figure 1).

The cellular distribution of CCRs also influences the progression of beta cell replication. In a series of studies, Fiaschi-Taesch et al. (2013a,b) observed that most cell cycle activators were preferentially located in the cytoplasm of human beta cells, where they were physically unable to drive cell cycle progression (Kassem et al., 2001; Fiaschi-Taesch et al., 2013a; Wang et al., 2015d). In the nucleus, only a few CCRs were detected, of which all were inhibitors: pRb, p57, and p21. When beta cell proliferation was enforced via overexpression of CDK6 and cyclin D3, a peculiar behavior of the cell cycle regulating molecules was observed: the activators CDK6, cyclin D3, and inhibitors p16, p21, p27 shuttled from the cytoplasm into the nucleus. Among other inhibitors, p57 decreased frequency of being in the nucleus, whereas p15, p18, and p19 did not change their localization, remaining in the cytoplasm.

The reasons for which cell cycle activators continue to be expressed in beta cells despite the low likelihood of division in these cells are poorly understood. One possible explanation (Wang et al., 2015d) is that CCRs have functions other than replication in beta cells, as has been shown in other cell types (Denicourt and Dowdy, 2004; Cánepa et al., 2007; Besson et al., 2008; Lim and Kaldis, 2013). For example, in post-mitotic neurons, cell cycle-related proteins are involved in migration, maturation, and synaptic plasticity (Herrup and Yang, 2007; Frank and Tsai, 2009; Frade and Ovejero-Benito, 2015). Another explanation for the constitutive expression of cell cycle activators is a need for the cells to be perceptively ready to replicate in case of elevated metabolic demands.

The cell cycle can be pushed toward mitosis by compounds called mitogens. The identification of new mitogens is the cornerstone of the development of novel treatment for diabetes based on expansion of the beta cell population.

### Proliferation Markers

In practice, the assessment of beta cell replication in the laboratory is often dependent on the immunohistochemical detection of cell cycle stages on the basis of proliferation markers. The most widely used replication marker is Ki67, a protein expressed during all phases of the cell cycle, except G0 (Sales Gil and Vagnarelli, 2018; Sun and Kaufman, 2018). Alternatively, thymidine analogs such as bromodeoxyuridine (BrdU) and 5-ethynyl-2′-deoxyuridine (EdU), can be used to detect cells in S phase, through the incorporation of these analogs into the newly synthesized DNA in place of thymidine (Sharma et al., 2019). Phospho-histone H3 (pHH3) is also widely used as a marker of proliferation. Histone 3 is phosphorylated in the late G2 phase, and this event can be used as a marker of the G2/M transition (Elmaci et al., 2018). A general concern is that although these markers reflect the cell cycle progression, they cannot unambiguously demonstrate whether it was completed with the cell division (Butler et al., 2007; Rieck et al., 2012; Tiwari et al., 2015). It is, therefore, essential to demonstrate actual changes in total beta cell number. Furthermore, these markers may be present during cell cycle-independent events, such as DNA damage and repair (Butler et al., 2007). DNA toxicity should therefore be assessed in addition to proliferation, for example, by phospho-γ-histone H2AX assay. In addition, evaluations of apoptosis rates, through terminal deoxynucleotidyl transferase dUTP nick-end labeling (TUNEL) assay could be used to rule out the possibility of cell number changes due to cell death (Wang et al., 2015d). Finally, it is important to bear in mind that since primary human beta cells are obtained postmortem, the general health of the donor before death and the islet isolation procedure may cause changes in the beta cells, thereby distorting the labeling of beta cells (Sullivan et al., 2015; Taniguchi et al., 2015).

Notably, most of the research on beta cell proliferation has been conducted on human islets dissociated into single beta cells, and seldom on intact, primary, human islets (Table 1). In intact islets, unlike in dispersed cells, the interconnections of beta cells between themselves and with other cell types, and the accompanying signaling are preserved. The microenvironment of the beta cells, including other endocrine pancreatic cells, as well as innervation and vasculature, is considered to substantially influence the proliferation of beta cells, and therefore the response of dispersed and intact islets might differ (Aamodt and Powers, 2017). Furthermore the 2D format of cell culture could alter the beta cell physiology and currently 3D formats are gaining increasing recognition due to a better mimicking of the natural cell arrangement occurring within the islets *in vivo* (Yang et al., 2020). The differences in the biology of dispersed beta cells vs. intact islets might impact the efforts to translate the *in vitro* findings into *in vivo*.

**TABLE 1:** Human beta cell mitogens and their potencies.

Mitogen	References	Experimental model	Labelling index (control vs mitogen), %				Cell cycle regulators		Signaling pathway
			Ki67	BrdU	EdU	pHH3	Activators	Inhibitors	
PDGF	Chen et al., 2011	*In vitro (p)*		0.5 vs. 3(effect only in juvenile)					PDGFR -> Erk activation -> Ezh2 induction
WS6	Shen et al., 2013	*In vitro (p)*	0.1 vs. 3						Inhibition of IKK -> NFkB translocation to the nucleus
		*In vitro (d)*			0.5 vs. 3				
	Boerner et al., 2015	*In vitro (p)*	0.3 vs. 0.8						
	Wang et al., 2015c	*In vitro (d)*	NS						
WS3	Dirice et al., 2016	*In vitro (d)*			NS				
Harmine	Wang et al., 2015c	*In vitro (d)*	0.1 vs. 1.2	0.1 vs. 1			CDK1, Cyclin A1, Cyclin E2, CDC25A, CDC25C, FOXM1, E2F1, E2F2, E2F7, E2F8	p15, p16, p57	Inhibition of DYRK1A -> NFAT translocation to the nucleus
	Aamodt et al., 2016	*In vitro (d)*	0 vs. 0.4						
	Dirice et al., 2016	*In vitro (d)*			0.5 vs. 2.5				
	Kumar et al., 2018	*In vitro (d)*	0.1 vs. 2.5						
	Wang P. et al., 2019	*In vitro (d)*	0 vs. 2.5	0 vs. 2.5		0 vs. 0.4	CDK1, Cyclin A1, Cyclin E2, CDC25A, FOXM1	p57	
		*In vivo*	0.5 vs. 1.2						
	Ackeifi et al., 2020a	*In vitro (d)*	0.1 vs. 3						
	Ackeifi et al., 2020b	*In vitro (d)*	0 vs. 2.5	0.2 vs. 2			CDK1, Cyclin A1, Cyclin A2, Cyclin E2, CDC25A, c-Myc, FOXM1	p15, p16, p57	
		*In vivo*	0.4 vs. 0.8						
	Rosado-Olivieri et al., 2020	*In vitro (sc)*			1 vs. 2.5				
INDY	Wang et al., 2015c	*In vitro (d)*	0 vs. 1.6				CDK1, Cyclin A1, Cyclin E2, CDC25A, CDC25C, FOXM1, E2F1, E2F2, E2F7, E2F8	p15, p16, p57	
	Wang P. et al., 2019	*In vitro (d)*	0.1 vs. 2.5						
	Ackeifi et al., 2020a	*In vitro (d)*	0.1 vs. 3						
5-IT	Dirice et al., 2016	*In vitro (d)*			0.1 vs. 5		CENPA, MCM2, MCM4, MCM5, CDC6, Cyclin B1, CDC20, TOP2A, RFC4		
		*In vivo*	0.1 vs. 0.4	0.1 vs. 0.4		0 vs. 0.2			
	Ackeifi et al., 2020a	*In vitro (d)*	0.1 vs. 2.8						
CC-401	Abdolazimi et al., 2018	*In vitro (d)*	0.2 vs. 0.7						
	Ackeifi et al., 2020a	*In vitro (d)*	0.1 vs. 0.8						
Leucettine-41	Wang P. et al., 2019	*In vitro (d)*	0.1 vs. 2						
	Ackeifi et al., 2020a	*In vitro (d)*	0.1 vs. 4.3						
TG003	Ackeifi et al., 2020a	*In vitro (d)*	0.1 vs. 2.3						
AZ191	Ackeifi et al., 2020a	*In vitro (d)*	0.1 vs. 0.5						
OTS167-derivatives	Allegretti et al., 2020	*In vitro (d)*	8-fold						
JAK3 inhibitor VI	Abdolazimi et al., 2018	*In vitro (d)*	0.2 vs. 0.6						
GNF7156	Shen et al., 2015	*In vitro (d)*			0 vs. 3				Inhibition of DYRK1A and GSK-3 beta -> NFAT translocation to the nucleus
GNF4877		*In vitro (d) In vitro (p)*			0 vs. 6 0.2 vs. 3				
		*In vivo*		1 vs. 3.5					
	Ackeifi et al., 2020a	*In vitro (d)*	0.1 vs. 4						
Tideglusib Chiron99021	Ackeifi et al., 2020a Ackeifi et al., 2020a Tsonkova et al., 2018	*In vitro (d) In vitro (d) In vitro (e)*	NS NS		0 vs. 20				Inhibition of GSK-3 beta -> NFAT translocation to the nucleus
PSN632408	Ansarullah et al., 2016	*In vivo*	0.7 vs. 2.5	1.5 vs. 6.3					GLP1R -> Ca^2+^ increase-> calcineurin increase-> NFAT translocation to the nucleus
GLP-1(7-36)amide	Ackeifi et al., 2020b	*In vitro (d)*	NS	NS			CDK4, Cyclin B3	p16, p18, p21	
Exendin-4	Aamodt et al., 2016	*In vitro (d)*	NS						
	Muhammad et al., 2017	*In vitro (p)*	2-fold						
	Dai et al., 2017	*In vivo*	juvenile:1.9 vs. 4adult:0.4 vs. 0.5				NFATC1, NFATC3, NFATC4, Cyclin A1, CDK1, FOXM1, EGR2, EGR3		
	Ackeifi et al., 2020b	*In vivo*	0.4 vs. 0.6						
OPG	Kondegowda et al., 2015	*In vitro (d)*		0.4 vs. 1.3					Inhibition of RANKL/RANK pathway ->GSK-3 beta inhibition, CREB-stimulation
DMB		*In vitro (d)*		0.4 vs. 0.8					
		*In vivo*		0 vs. 0.1					
SerpinB1 Silvestat	El Ouaamari et al., 2016	*In vitro (p) In vitro (p) In vivo*	0.01 vs. 0.05 0.1 vs. 0.05 0 vs. 0.1						Inhibition of GSK-3 beta, alteration of MAPK, PRKAR2B-> NFAT translocation to the nucleus
Glucose	Stamateris et al., 2016	*In vitro (d)*		Up to 1.2					Activation of mTOR pathway
SB431542	Dhawan et al., 2016	*In vivo*	0.2 vs. 0.5	0 vs. 0.3		0.05 vs. 0.1		p16	Inhibition of TGF-beta pathway
ALKV Inh. II	Abdolazimi et al., 2018	*In vitro (d)*	NS						
D4476		*In vitro (d)*	NS						
SB431542	Hakonen et al., 2018	*In vitro (p)*			NS				
LY364947	Wang P. et al., 2019	*In vitro (d)*	NS	NS		NS		p15, p16, p21, p57	
GW788388		*In vivo*	0.5 vs. 1						
GABA	Purwana et al., 2014	*In vivo*		0.5 vs. 2.3					GABA_*A*_/_*B*_R -> Ca^2+^ increase -> Activation of PI3K/Akt pathway, CREB activation
	Aamodt et al., 2016	*In vitro (d)*	NS						
	Prud’homme et al., 2017	*In vitro (p)*	0.5 vs. 3.2						
	Tian et al., 2017a	*In vitro (p)*		up to 1.2					
Lesogaberan	Tian et al., 2017b	*In vitro (p)*		2.7-fold					
		*In vivo*	0.5 vs. 0.9	0.9 vs. 2.3					
LIF	Rosado-Olivieri et al., 2020	*In vitro (sc)*			1 vs. 1.5		Cyclin A2, Cyclin B1, Cyclin B2, Cyclin E2, CDK2, CDK4	p16, p18, p19	Activation of LIF pathway:LIFR-STAT3-CEBPD activation
		*In vitro (d)*					Cyclin B1, Cyclin B2, Cyclin D1, Cyclin E2, CDK2		
		*In vivo*			0.4 vs. 1.5				
MANF	Hakonen et al., 2018	*In vitro (p)*			NS				Inhibition of NF-κB pathway
MI-2	Chamberlain et al., 2014	*In vitro (p)*			0 vs. 0.6				Inhibition of menin -> activation of MAPK
MI-2-2	Muhammad et al., 2017	*In vitro (p)*	2.3-fold						

*NS, not significant.*

*For each mitogen, the available information is:*

*– Mitogen applied (column 1);*

*– Reference (column 2);*

*– Model used (column 3): pstands for primary islets; d stands for dispersed beta cells; sc stands for human stem cell-derived beta cells; e stands for EndoC-βH1 cell line; and in vivo implies human beta cells/islets engrafted into mouse;*

*– Proliferation index (columns 4–7). The values are presented as index from control beta cells vs. the cells treated with mitogens; by default, the values are given in percentage and for the cases with different units, the units are indicated (for example, fold change). Depending on the proliferation markers assessed (Ki67, BrdU, EdU, or pHH3), the values are situated in the column dedicated for the corresponding marker;*

*– Effect on cell cycle regulators: upregulated activators (column 8) and downregulated inhibitors (column 9) in response to mitogen treatment;*

*– Signaling pathway through which the mitogen acts (column 10).*

It should be stressed that evaluations of beta cell proliferation rates and comparisons between studies are generally hampered by differences in the choice of proliferation markers between research groups. Each proliferation marker has its own characteristic level of detectability, and comparisons between studies using different markers (Table 1), and quantification methods (i.e., software for image analysis, flow cytometry or quantitative PCR) are therefore difficult. Furthermore, the beta cell replication index may differ between regions of the pancreas region, such as the head and the tail. It is therefore important to standardize laboratory protocols for the evaluation of beta cell proliferation.

### Beta Cell Proliferation in Humans and Mice

The search for agents boosting beta cell proliferation led to the identification of numerous mitogens in rodents, including glucose, insulin, insulin-like growth factor I (IGF-1), hepatocyte growth factor (HGF), incretins (glucagon-like peptide-1 (GLP-1), exendin-4, liraglutide, glucose-dependent insulinotropic polypeptide (GIP), lactogens [growth hormone (GH), prolactin (PRL), placental lactogen (PL)], betacellulin (BTC), epiregulin, platelet-derived growth factor (PDGF), leptin, estrogen, progesterone, parathyroid hormone–related protein (PTHrP), TGF-beta inhibitors, adenosine kinase signaling inhibitor, serotonin, gamma-aminobutyric acid (GABA), gastrin, osteocalcin, and thyroid hormone T3 (Vasavada et al., 2006; Kulkarni et al., 2012; Bernal-Mizrachi et al., 2014; Stewart et al., 2015; Wang et al., 2015d; Baeyens et al., 2018; Jiang et al., 2018). Extensive research resulted in the identification of a plethora of downstream targets for these mitogens (Kulkarni et al., 2012; Bernal-Mizrachi et al., 2014; Stewart et al., 2015). These mitogens significantly increased rodent beta cell proliferation, and similar outcomes were expected for human beta cells (Bonner-Weir and Weir, 2005; Vasavada et al., 2006). However, the expectations were rapidly dispelled when attempts were made to translate these findings from rodents to humans, as majority of mitogens identified in rodents failed to induce similar effects in humans (Parnaud et al., 2008; Kulkarni et al., 2012; Skyler, 2018).

The general life-long replication dynamics of beta cells appears to be similar in humans and rodents, with replication rates highest in the early postnatal period and declining with age. However, the magnitudes of these replication rates differ considerably between humans and rodents. In humans, beta cell replication rates are in the range of a tenth of a percent to a couple of percent, whereas, in rodents, they may reach 20%, subsequently declining to ∼2% in early adulthood (Finegood et al., 1995; Kulkarni et al., 2012; Wang et al., 2015d). Moreover, the cell cycle machinery of beta cells differs between rodents and humans. For example, CDK6 is absent in rodents, but indispensable for the proliferation of human beta cells (Fiaschi-Taesch et al., 2009, 2013b). Cyclin D3, which is not essential in rodents, plays a crucial role in the replication of human beta cells (Köhler et al., 2011; Fiaschi-Taesch et al., 2013b). Conversely, Cyclin D2, which is essential in rodents, is expressed only at very low levels, if at all, in human beta cells (Fiaschi-Taesch et al., 2010; Kulkarni et al., 2012). In addition, higher replication rate of rodent beta cells could be attributed to the specificities of the rodent organism for example higher metabolism rates (Gorbunova et al., 2008). These differences may have contributed to the disappointing results obtained for rodent mitogens in humans, leading to a growing realization that there are critical differences in beta cell proliferation between rodents and humans.

### Mitogens for Human Beta Cells

A number of human beta cell mitogens have been discovered over the last decade (Table 1). Interestingly, many of these mitogens, including harmine, GNF4877, INDY, 5-IT, CC-401, and OTS167, have similar mechanisms of action – the inhibition of dual-specificity tyrosine-phosphorylation regulated kinase 1A (DYRK1A) (Wang et al., 2015b,c; Ackeifi et al., 2020a), highlighting the importance of this protein in beta cell proliferation. DYRK1A phosphorylates nuclear factor of activated T-cells transcription factors (NFATs) and keeps NFATs inactive in the cytoplasm. DYRK1A inactivation allows NFATs to be translocated to the nucleus, where they activate the expression of cell cycle entry-promoting genes: Forkhead Box M1 (FOXM1), cyclin E1, cyclin A2, and CDK1, and repress the expression of inhibitors, such as p57, p15, and p16 (Goodyer et al., 2012; Karakose et al., 2018). In parallel, DYRK1A inhibits beta cell proliferation by stabilizing the p27 inhibitor and the DREAM (dimerization partner, RB-like E2F and multi-vulval class B) protein complex (Abdolazimi et al., 2018), leading to an inhibition of cell cycle-promoting transcription factors, such as c-Myc, thereby maintaining cells in G0 phase (Sadasivam and DeCaprio, 2013).

The mitogenic potential of DYRK1A inhibitors is highly variable. It has been suggested that GNF4877 and 5-IT are more effective than harmine, as they produce beta cell mitogenic effects similar to or stronger than those of harmine, but at lower doses (Shen et al., 2015; Dirice et al., 2016; Ackeifi et al., 2020a). *In vitro*, harmine has been shown to induce the proliferation of up to 3% of human beta cells, whereas untreated dispersed beta cells have a proliferation rate of only 0.1% (Ackeifi et al., 2020a). In turn, GNF4877 increases human beta cell proliferation to up to 4% at a dose one tenth that of harmine, as indicated by Ki67^+^/Ins^+^ cell quantification (Ackeifi et al., 2020a). The greater mitogenic potential of GNF4877 and 5-IT than of harmine may result from effects on a wider range of signaling pathways. In addition to DYRK1A, GNF4877 inhibits glycogen synthase kinase-3 beta (GSK-3 beta), This kinase negatively controls beta cell replication via the retention of NFAT in the cytoplasm, in a mechanism similar to that observed for DYRK1A (Shen et al., 2015). Notably, one of DYRK1A inhibitors common target is DYRK1B kinase which can compensate for DYRK1A depletion (Ackeifi et al., 2020a). The diverse targets of the DYRKA1-inhibiting mitogens align with the diversity of downstream cell cycle-associated genes (Table 1).

Signaling pathways other than the DYRK1A-NFAT pathway have been shown to play an important role in beta cell replication. For example, TGF-beta inhibitors, including GW788388, SB431542, and LY364947, suppress TGF-beta receptor-Smad signaling, decreasing the expression of p16 (Dhawan et al., 2016), p15, p21, and p57 (Wang P. et al., 2019). Leukemia inhibitory factor (LIF), a newly identified mitogen of hPSC-derived beta cells (SC-beta), acts through STAT3 and CEBPD signaling and induces the expression of many cell cycle activators, having the highest effect on Cyclin E2 (Rosado-Olivieri et al., 2020; Table 1) in both SC-beta cells and human primary islets. GABA acts through the binding to the GABA receptors on human beta cells, leading to an influx of Ca^2+^ and activation of the Ca^2+^-dependent PI3K/Akt and CREB signaling pathways responsible for beta cell proliferation and survival (Wang Q. et al., 2019). The signaling pathways involved in human beta cell proliferation have been described in more detail elsewhere (Shirakawa and Kulkarni, 2016; Karakose et al., 2018; Basile et al., 2019).

Most beta cell mitogens also induce proliferation of alpha cells and other endocrine cells (Gutierrez et al., 2018; Yang et al., 2020), which further limits their suitability for beta cell regeneration in diabetes. For example, harmine was shown also to induce proliferation of alpha cells (Shirakawa and Kulkarni, 2016; Kumar et al., 2018). Importantly, the combinations of mitogens may have a greater and more specific effect on beta cell proliferation than individual molecules. Manipulation of the expression levels of CCRs will also very likely affect *in vivo* other cells than beta cells. Thus, this approach might serve as an experimental platform to better understand signals governing beta cell replication rather than a translational approach.

## The Synergy of Human Beta Cell Mitogens

### The Synergy of Cell Cycle Regulators

In drug development, synergy implies that a combination of two or more pharmacological agents has a therapeutic effect greater than that of each agent applied individually, or greater than the simple additive effect of the combined agents (Foucquier and Guedj, 2015). Two drugs may be considered to act in synergy if they, for example, inhibit two proteins active in parallel pathways essential for an observed phenotype (*parallel pathway inhibition model*), or if one drug improves the availability of the second drug in the target cell by promoting the entry of the second drug into the cell or decreasing its degradation (*bioavailability model*) (Cokol et al., 2011). Thus, the simultaneous use of several pharmacological agents may influence on a larger number of signaling pathways or decrease the dose of each agent required, thereby increasing efficacy whilst decreasing adverse effects and toxicity. These key benefits could render the combinational therapies superior over the monotherapies (Breitinger, 2012; Foucquier and Guedj, 2015).

The rationale for a synergic approach in studies aiming to increase the proliferation of human beta cells can be traced back to a time when no reliable mitogens had yet been discovered. Genetic manipulations, such as viral vector-mediated gene overexpression or silencing, were used to investigate the cell cycle machinery for beta cell replication. A series of studies indicated that multiple targeting would be required to induce beta cell proliferation more effectively. For example, Cozar-Castellano et al. (2004) showed that even though the simultaneous adenoviral overexpression of CDK4 and cyclin D1 did not result in a proliferation rate higher than that achieved by the overexpression of each of these molecules alone, it did lead to a higher level of pRb phosphorylation than the overexpression of each protein alone (Cozar-Castellano et al., 2004). Fiaschi-Taesch et al. (2009) showed that the combination of cyclin D1 and CDK6 boosted beta cell replication effectively, resulting in a BrdU labeling index of 13% in undispersed human beta cells (Fiaschi-Taesch et al., 2009). Another combination, cyclin D3 and CDK6, was subsequently shown to yield a BrdU labeling index of 15% (Fiaschi-Taesch et al., 2010). Kondegowda et al. (2010) overexpressed late G1/S complex, cyclin E, and CDK2, which led to a 16-fold increase in BrdU labeling index relative to control (Kondegowda et al., 2010). Finally, Tiwari et al. (2015) overexpressed the early and late cyclin/CDK complexes — CDK6, cyclin D3, CDK1, and cyclin E — simultaneously, and showed that this yielded an impressive proliferation rate: 43% for BrdU and 54% for Ki67 indexes. However, this high replication rate was accompanied by a 25% increase in DNA damage, indicating toxicity due to viral vector overload. A decrease in the multiplicity of infection to overcome this toxicity resulted in rates of 19 and 35% for BrdU and Ki67 labeling, respectively. This was still higher than any of the two, early and late, complexes alone. Importantly, the increase in cell proliferation was confirmed by the actual increase in beta cell number. The authors concluded that the early and late complexes enter the cell cycle independently and act in a complementary manner (Tiwari et al., 2015). The authors did not use mitogens, but they hypothesized that identification of the mitogens able to trigger the nuclear translocation of both the early and late complexes might be needed for effective therapeutic approaches for diabetes based on beta cell replication. In turn, Robitaille et al. (2016) targeted known cell cycle inhibitors with short hairpin RNAs (shRNAs) and found that the silencing of p18 and p21 together resulted in higher rates of proliferation (∼15% of beta cells were EdU^+^), than the silencing of either p18 (∼4.5% EdU^+^) or p21 (∼8% EdU^+^) alone (Robitaille et al., 2016), while only ∼1.5% cells were EdU^+^ among the untreated beta cells.

These combinatorial strategies yielded the highest rates of replication and therefore demonstrated the weightiness of synergy in research on beta cell replication.

### Mitogenic Synergy

With the identification of the first human mitogens, most studies initially focused on the properties of individual mitogens, without consideration of their synergic potential. However, some applications involving two mitogens were reported (Table 2). For example, Liu et al. (2009) showed that the combination of glucose and a GSK-3 beta inhibitor (lithium or 1-Akp) synergically promoted beta cell proliferation (Liu et al., 2009). Chamberlain et al. (2014) found that exendin-4 was unable to stimulate the proliferation of human beta cells when used alone, but that such stimulation was observed when exendin-4 was used in combination with MI-2, the inhibitor of the menin-histone methyltransferase MLL interaction (Chamberlain et al., 2014). Hakonen et al. (2018) reported that a combination of mesencephalic astrocyte-derived neurotrophic factor (MANF) and a TGF-beta inhibitor, SB431542, increased beta cell proliferation, whereas no such increase was observed when either of these agents were used alone. Alprazolam, a positive allosteric modulator (PAM) acting via the GABA receptor, markedly increases the ability of GABA to promote beta cell proliferation (Tian et al., 2017b). However, not every mitogen combination was able to improve beta cell proliferation. For instance, Caballero et al. (2014) showed that gastrin or exendin-4 alone, or a combination of gastrin and exendin-4, or gastrin and EGF, did not promote beta cell replication in mice engrafted with human islets (Caballero et al., 2014). Muhammad et al. (2017) reported that the Ki67 labeling of ins^+^ cells in islets treated with a combination of MI2-2 and exendin-4 was no better than that achieved with either of these agents used singly (Muhammad et al., 2017). Similarly, a lack of synergic effect was observed for a combination of GABA and Klotho (Prud’homme et al., 2017).

**TABLE 2:** Studies on synergic action of compounds with human beta cell mitogenic properties.

Study Mitogen combination	Model	Effect on proliferation		
Liu et al., 2009				
Glucose + LiCl or 1-Akp (GSK-3 inhibitors)	*In vitro (p)*	Ki67 immunostaining, fold		Synergic increase
		Glucose	1.8	
		Glucose + LiCl	3.4	
		Glucose + 1-Akp	2.5	
		BrdU immunostaining, fold		
		Glucose	1.8	
		Glucose + LiCl	4	
		Glucose + 1-Akp	3	
Caballero et al., 2014				
Gastrin + Exendin-4, Gastrin + EGF	*In vivo*	Ki67 immunostaining, %		No significant synergic effect
		Control	0.3	
		Gastrin	0.4	
		Exendin-4	0.3	
		Gastrin + Exendin-4	0.4	
		Gastrin + EGF	0.2	
Chamberlain et al., 2014				
Exendin-4 + MI-2 (Menin–MLL interaction inhibitor)	*In vitro (p)*	Edu immunostaining, %		Synergic increase
		Control	0	
		MI-2	0.6	
		Ex-4	0	
		MI-2 + Exendin-4	1.3	
Tian et al., 2017a				
GABA + alprazolam	*In vitro (p)*	^3^H-thymidine incorporation, fold		Synergic increase
		GABA	1.8	
		GABA + alprazolam	2.2	
Muhammad et al., 2017				
Exendin-4 + MI-2-2 (Menin inhibitor)	*In vitro (p)*	Ki67 mRNA expression, fold		No significant synergic effect
		MI2-2	2.3	
		Exendin-4	2	
		MI2-2 + Exendin-4	1.8	
Prud’homme et al., 2017				
GABA + Klotho protein	*In vitro (p)*	Ki67 immunostaining, %		No significant synergic effect
		Control	0.5	
		GABA	3.2	
		Klotho	2.5	
		GABA + Klotho	3.5	
Hakonen et al., 2018				
MANF + SB431542 (TGF-beta inhibitor)	*In vitro (p)*	Edu immunostaining, %		Synergic increase
		Control	0.6	
		SB431542	0.7	
		MANF	0.5	
		MANF + SB431542	1.3	
Abdolazimi et al., 2018				
CC-401 (DYRK1A inhibitor) + GSK-3 beta or ALK5/TGF-beta inhibitors	*In vitro (d)*	Ki67 immunostaining, %		Synergic increase
		Control	0.5	
		CC-401	1	
		ALK5/TGF-beta inh	0.5	
		CC-401 + ALK5/TGF-beta inh	4	
Wang P. et al., 2019				
Harmine (DYRK1A inhibitor) + LY364947 or GW788388 (TGF-beta inhibitors)	*In vitro (d)*	Ki67 immunostaining, %		Synergic increase
		Control	0	
		Harmine	2	
		LY364947	NS	
		LY364947 + Harmine	5–8	
	*In vivo*	Control	0.5	
		Harmine	1.2	
		GW788388	1.1	
		Harmine + GW788388	1.7	
Rosado-Olivieri et al., 2020				
LIF+ Harmine + LY364947 (TGF-beta inhibitor)	*In vitro (sc)*	Edu immunostaining, %		Synergic increase
		Control	1	
		LIF	1.5	
		Harmine	2.5	
		Harmine + LY364947	3.8	
		Harmine + LY364947 + LIF	5	
Ackeifi et al., 2020a				
Harmine + Tideglusib or Chiron99021 (GSK-3 beta inhibitors)	*In vitro (d)*	Ki67 immunostaining, %		Synergic increase
		Control	0	
		Tideglusib	0	
		Chiron99021	0	
		Harmine	2	
		Tideglusib + Harmine	3	
		Chiron99021 + Harmine	3.5	
Ackeifi et al., 2020b				
Harmine + GLP1	*In vitro (d)*	Ki67 immunostaining, %		Synergic increase
		Control	0	
		GLP-1	0	
		Harmine	2.5	
		GLP-1 + Harmine	6	
	*In vivo*	Control	0.4	
		Exendin-4 (GLP-1 analogue)	0.5	
		Harmine	0.8	
		Exendin-4 + Harmine	1.1	

*For each study, information is given:*

*– The mitogen combination(s) (column 1);*

*– The model used (column 2): pstands for primary islets, d stands for dispersed beta cells, sc stands for human stem cell-derived beta cells, and in vivo implies human beta cells/islets engrafted into mouse;*

*– The proliferation assay (column 3);*

*– The effects of the treatment in a form of proliferation indices for the single mitogens, and mitogen combinations (columns 3–5).*

**FIGURE 2:**
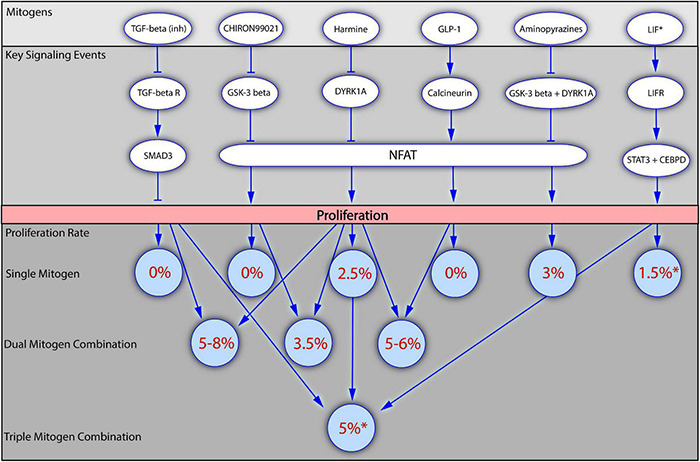
Mitogenic synergy in human beta cell proliferation. The figure presents how application of mitogens alone or in combinations influences the beta cell proliferation. Several of the most potent mitogenic compounds are depicted in the top block, together with the key signaling events through which they regulate beta cell proliferation (the block below). The bottom block of the figure demonstrates how the values of beta cell proliferation rates increase upon the application of dual or triple mitogen combinations in comparison to the effects of the individual mitogens. Numbers in brackets indicate the literature references. *For all the mitogens, except LIF, the values of proliferation rates were obtained with the staining for Ki67 presence, and are therefore suitable for comparison. For LIF the proliferation rate has been obtained with the EdU incorporation assay. LIF is included into the scheme as the first mitogen described in a triple combination (LIF + Harmine + TGF-beta inhibitor).

Recently, several studies deliberately focusing on the synergic effects of mitogens on human beta cell proliferation have been reported (Abdolazimi et al., 2018; Wang P. et al., 2019; Ackeifi et al., 2020b; Rosado-Olivieri et al., 2020; Figure 2), confirming that the application of a combination of several mitogens has a greater effect than each of the mitogens applied alone. The use of combinations of known mitogens allowed to improve the rate of proliferation *in vitro* to 8%, which is even higher than the rate of beta cell proliferation generally observed during the early postnatal period.

Abdolazimi et al. (2018) used high-content screening to identify 254 compounds stimulating beta cell replication. A strong synergic effect on rat beta cells was observed following the application of a novel mitogen, CC-401, in combinations with other mitogens, reaching the Ki67 index value of 31%. The majority of these combinations were not effective on human beta cell proliferation, confirming once again the biological differences in beta cell replication between rodents and humans. Two combinations, however, increased human beta cell proliferation: CC-401 combined with an inhibitor of GSK-3 beta (Chiron99021) or ALK5/TGF-beta (D4476 and ALK5 inhibitor II) increased replication in undispersed human beta cells to a greater extent than CC-401 alone. The results for Chiron99021 were inconsistent, but the combination of CC-401 and D4476 resulted in 2% of ins^+^ cells displaying Ki67 labeling, with almost 4% labeling observed with the combination of CC-401 and ALK5 inhibitor II. By contrast, the application of CC-401 alone resulted in the labeling of 1% of beta cells with Ki67, versus <0.5% in DMSO-treated controls (Abdolazimi et al., 2018).

Wang P. et al. (2019) investigated the effect of a combination of harmine and TGF-beta inhibitors (LY364947, GW788388, or SB43154) on human islet proliferation. Proliferation was observed in 2% of harmine-treated beta cells, as shown by determining levels of Ki67^+^/ins^+^ cells, whereas TGF-beta inhibitors had no effect on beta cell replication. The simultaneous application of the two mitogens increased beta cell proliferation rates to 6%, and even to 18% in some individual batches of islets. The increase in beta cell replication was confirmed by an actual increase in cell numbers. The effect was verified in an *in vivo* model: non-obese diabetic severe combined immunodeficient (NOD-SCID) mice in which human islets were implanted received intraperitoneal injections of a single mitogen or the combination of mitogens. The harmine and TGF-beta inhibitor combination was again superior, with 1.7% Ki67^+^ beta cells, the highest proportion of proliferating cells yet achieved in the mouse-based *in vivo* human model. Investigations of the impact of mitogens on the cell cycle showed that harmine acted through the inhibition of DYRK1A, stimulating cell cycle activators, such as CDK1, cyclin A1, cyclin E2, and CDC25A, with limited effect on cell cycle inhibitors other than p57. By contrast, TGF-beta inhibition alone had little effect on cell cycle activators, but strongly repressed the expression of cell cycle inhibitors, such as p15, p21, and p57. Proliferation was therefore increased by combining the ability of harmine to induce cell cycle activators with the ability of TGF-beta inhibitors to block cell cycle repressors (Wang P. et al., 2019).

Ackeifi et al. (2020b) investigated the effect of a combination of GLP-1 [GLP-1(7-36)amide] and harmine on the proliferation of human primary islets. Harmine treatment resulted in 2% Ki67/ins labeling, whereas GLP-1 had no appreciable effect. Combined treatment resulted in the Ki67/ins labeling of 5% of the total beta cells. In mice engrafted with human islets, treatment with this combination increased the Ki67/ins labeling index from the control value of <0.4–1%; this combination was more effective than any of the single treatments. Finally, the effect of a combination of harmine and GLP-1 was investigated in a diabetic mouse engrafted with human islets. Mice treated with one of these compounds alone remained diabetic, whereas glucose concentration returned to near-normal levels in mice treated with the combination of mitogens. Examination of the cell cycle events revealed that harmine induced expression of the cell cycle activators CDK1 and cyclin A1, confirming previous findings reported by Wang Q. et al. (2019). Harmine also decreased the expression of p15, p16, and p57. In turn, GLP1 had little effect on cell cycle activators but repressed the expression of p16, p18, and p21. The repression of cell cycle inhibitors by GLP-1 made the key contribution to the overall synergic effect, leading to an increase in beta cell proliferation (Ackeifi et al., 2020b).

The possible synergy of harmine and TGF-beta inhibitor with one more mitogen, LIF, in promoting SC-beta cell proliferation has also been tested. The combination of these three mitogens resulted in an EdU labeling index of 5%, versus ∼1% in DMSO-treated controls. This three-compound combination was more effective than the two-compound combination of harmine + TGF-beta inhibitor, which induced proliferation in 3% of SC-beta cells (Rosado-Olivieri et al., 2020). These results independently confirmed the efficacy of the previously reported harmine and TGF-beta-inhibitor combination and suggested that the powerful effect of this combination could be enhanced even further by addition of other mitogens, providing a rationale for seeking even more robust combinations.

### Fine-Tuning of Synergy Between Mitogens

All these studies demonstrated that the simultaneous application of several mitogens could give unprecedented high proliferation rates, higher than those obtained with any of the individual mitogens used alone. They also provided important insights into the general mechanisms of beta cell proliferation. For instance, treatments with multiple mitogens increase the reentry of beta cells into the cell cycle by widening the number of the downstream targets, rather than through shared downstream targets. Interestingly, the combinatory effect was not limited to a particular mitogen, but could be reproduced with many different mitogens with similar modes of action (e.g., inhibitors of DYRK1A or TGF-beta), through a class effect. Peculiarly, some compounds, such as TGF-beta inhibitors, do not act as mitogens when applied alone, their mitogenic effects instead being revealed only when they are applied in combinations with other mitogens.

The use of several mitogens to regulate the cell cycle can result in beta cell proliferation rates of up to 8%, which is still much lower than the rate obtained by directly manipulating the expression of multiple cell cycle regulatory genes. As described above, the viral mediated upregulation of cyclins/CDKs and downregulation of cell cycle inhibitors yields up to 35% Ki67 labeling (Tiwari et al., 2015). It is reasonable to assume that the induction of beta cell replication by mitogens may be weaker due to critical, yet undefined signaling events. A comprehensive examination of the downstream mitogen-mediated events is, therefore, indispensable: it might reveal additional targets, and consequently make it possible to establish more effective mitogen combinations. Pursuing this perspective, in Table 1 we collected the information about the cell cycle-associated genes targeted by various mitogens.

Some mitogens, such as the TGF-beta inhibitors or GLP-1, appear to act predominantly or exclusively through the targeting cell cycle inhibitors, whereas others, such as aminopyrazines, regulate cell cycle activators. Meanwhile, harmine affects both cell cycle activators and inhibitors, which could explain the powerful effects of this mitogen on beta cell proliferation. Moreover, in addition to inhibiting DYRK1A, harmine has been reported to downregulate SMAD proteins, which are intermediates of the TGF-beta pathway (Wang P. et al., 2019), potentially accounting for the ability of harmine to downregulate p-inhibitors.

Noteworthy, specialist methods and algorithms have been developed for the discovery and assessment of drug combination effects (Cokol et al., 2011; Chen et al., 2015; Foucquier and Guedj, 2015; Tang et al., 2015; Meyer et al., 2020). Conceptually, once combinations of mitogens for inducing beta cell proliferation have been established and described, the most promising of those combinations should be investigated in greater depth, to unravel the key mechanisms of the powerful mitogenic activities observed.

Comprehensive elucidation of the signaling events involved in beta replication will require the use of more complex and high-throughput techniques, such as scRNA-seq.

## Contribution of RNA-Sequencing Technologies to Research on Beta Cell Proliferation

### Advantages of Transcriptomic Technologies in Beta Cell Research

Transcriptomic techniques have made it possible to define bulk- and scRNA-seq-based beta cell molecular signatures (Li et al., 2016; Segerstolpe et al., 2016; Xin et al., 2018) and to identify the rare proliferating beta cells among the abundant quiescent beta cells (Klochendler et al., 2016; Rosado-Olivieri et al., 2020). A detailed examination of the gene expression profiles in proliferating cells can then accelerate the identification of key processes endowing these cells with higher proliferation capacities.

For instance, Klochendler et al. (2016) generated transgenic mice expressing GFP fused to the N-terminus of cyclin B1, to identify and enrich the proportion of proliferating beta cells. Bulk RNA-seq was then performed on these proliferating cells and on the quiescent cells. As expected, the RNA-seq analysis showed an enrichment in the expression of genes associated with DNA synthesis and repair, mitosis, and chromatin remodeling in the cyclin B1-GFP^+^ beta cells relative to quiescent cells. Furthermore, an inverse correlation was found between the expression of genes associated with beta cell function (principally insulin processing, granule formation, and secretion) and that of genes associated with proliferation. The authors investigated whether the expression of beta cell function-associated genes was downregulated in proliferating beta cells, by studying the transcriptome of EndoC-βH2 human beta cell line, which display conditional proliferation (Scharfmann et al., 2014). In EndoC-βH2 cells, exit from the cell cycle was accompanied by the activation of genes associated with beta cell function, which overlapped with genes repressed in replicating cyclin B1-GFP^+^ mouse beta cells. A similar trend of negative correlation between proliferation and maturity was reported by Puri et al. (2018), who primed mouse beta cells to proliferate via upregulation of c-Myc expression to non-oncogenic levels. The primed cells exhibited a profile of immature beta cells, namely immature insulin processing, packaging, and impaired secretory response. Comparative RNA-seq analysis of control against the c-Myc-induced proliferating beta cells revealed upregulation of the genes associated with immature profile, and downregulation of the genes associated with cell maturation and regulation of insulin secretion (Puri et al., 2018). Thus, decreases in the expression of genes controlling beta cell function in proliferating beta cells may be conserved between mice and humans.

scRNA-seq provides even wider opportunities for the analysis of beta cell replication than bulk RNA-seq (Wang and Kaestner, 2019). Pseudotime analyses of single-cell transcriptomes can be performed to explore cell cycle dynamics. This method orders and subclusters individual cells on a virtual axis on the basis of the degree of similarity between their transcriptomes, making it possible to detect cellular dynamics, such as progression through the cell cycle or cell maturation (Bendall et al., 2014; Trapnell et al., 2014; Zeng et al., 2017; Wang and Kaestner, 2019). The pseudotime ordering of murine beta cells from postnatal days 1 – 28 (P1 – 28) showed that the mature beta cell profile became stronger with age and was accompanied by a decrease in the expression of genes associated with the cell cycle and proliferation (i.e., MKI67, CDK4, RFC2, and MCM3). Maturation was also positively correlated with decreases in the expression of genes associated with the transport of amino acids, which are indispensable for protein and nucleotide synthesis during cell proliferation. This observation led to the hypothesis that the decline in proliferation may, to some extent, result from a decrease in amino acid availability in the plasma of aging mice. Indeed, the *in vitro* supplementation of islets from P28 mice with serine, tyrosine, and nucleotides increased beta cell proliferation (Zeng et al., 2017). In another study, by Tatsuoka et al. (2020), pseudotime analysis of cell cycle progression was performed in mice with pancreatectomy. The beta cells were clustered according to their position in the cell cycle. Interestingly, during progression through the cell cycle, genes associated with UPR (ATF6, HSPA5) and the inhibition of proliferation (p-inhibitors, the TP53 gene and its targets) displayed an increase in expression, presumably mirroring anti-oncogene regulation of the cell cycle (Tatsuoka et al., 2020).

For human beta cells, pseudotime ordering has been used to investigate the relationship between cell cycle progression and insulin production (Xin et al., 2018). Most proliferating beta cells clustered together and presented low levels of insulin and high levels of UPR gene expression (INS^lo^UPR^hi^). These observations resemble those reported by Tatsuoka et al. (2020) for mice. Xin and coworkers also found that INS^lo^UPR^hi^ beta cells had higher levels of expression for genes involved in energy metabolism than INS^lo^UPR^lo^, and INS^hi^UPR^lo^ cells, suggesting that INS^lo^UPR^hi^ beta cells use more energy than cells with higher insulin levels (Xin et al., 2018). Notably, among the transcription factors found to be enriched in this state were those regulating embryonic stem cell self-renewal and proliferation, such as NR0B1 and ZNF143.

The RNA-seq was also applied to investigate the molecular changes associated with rodent beta cell proliferation during pregnancy. For example, RNA-seq data analysis pointed to prolactin and serotonin signaling responsible for the regulation of beta cell expansion during pregnancy in mice (Kim et al., 2010; Sisino et al., 2017; Shrivastava et al., 2021).

Examination of the beta cell transcriptome might also lead to the discovery of previously unknown mitogenic compounds. Rosado-Olivieri et al. (2019) demonstrated that overexpression of the Hippo pathway effector Yes-associated protein 1 (YAP) induces cell cycle reentry in SC-beta cells. A scRNA-seq analysis of YAP-overexpressing beta cells revealed an enrichment in the Hippo and LIF signaling pathways. The treatment of SC-beta cells with the LIF ligand was subsequently shown to increase replication, demonstrating that LIF functions as a beta cell mitogen. The YAP-enriched human SC-beta cells also displayed a downregulation of genes involved in primary beta cell functions, including insulin secretion, again suggesting that beta cell replication provokes a reversion of the cells to immaturity. Furthermore, the authors also identified a new beta cell subpopulation characterized by LIF receptor (LIFR) expression (Rosado-Olivieri et al., 2020); these cells represent an example of the replication-competent subpopulations within the total beta cell pool.

### Proliferating Subpopulations of Beta Cells

The beta cell population is heterogeneous, with differences in gene expression between individual beta cells (for a comprehensive review see Pipeleers et al., 1994, 2017; Bengtsson et al., 2005; Roscioni et al., 2016; Gutierrez et al., 2017; Benninger and Hodson, 2018). Cells with close similarity of features such as transcriptome or functioning could be recognized as distinct beta cell subpopulations. The subpopulations vary in frequency within the total beta cell population, depending on the state of the organism such as age, disease, pregnancy or obesity (Dorrell et al., 2016; Zeng et al., 2017). Accordingly, in mouse models by the means of scRNA-seq it was demonstrated that proliferative beta cells are more abundant during early life, subsequently becoming less abundant in aging mice (Zeng et al., 2017).

Several different beta cell subpopulations with a higher proliferation capacity have been described. The LIFR^+^ beta cell subpopulation in humans (Rosado-Olivieri et al., 2020) and beta cells lacking the Flattop (Fltp), an effector of Wnt/Planar cell polarity signaling, in mice have been reported to be proliferation-competent. Fltp^–^ cells, which account for ∼20% of total beta cells, show stronger labeling for proliferation markers and weaker p27 expression (Bader et al., 2016). It is tempting to assume that enrichment in replication-competent subpopulations, such as LIFR^+^ cells, and the treatment of these cells with mitogens would result in more robust beta cell expansion, because the mitogenic effect would not be diluted over the entire beta cell population. However, care is required when considering this perspective. First, it has been suggested that different subpopulations of beta cells perform different functions (Gutierrez et al., 2017), as best exemplified by the discovery of the so-called “hub” beta cells (Johnston et al., 2016). These hub cells are relatively rare beta cells (<10% of total beta cells) acquired with pacemaker-like properties that orchestrate the activities of the remaining “follower” beta cells during the insulin response (Johnston et al., 2016). Second, as described above, there is a growing body of evidence to suggest that higher levels of proliferation are associated with a relatively immature beta cell profile both in mice (Bader et al., 2016; Klochendler et al., 2016; Zeng et al., 2017; Puri et al., 2018; Zhang et al., 2018) and humans (Klochendler et al., 2016; Xin et al., 2018; Rosado-Olivieri et al., 2020). Hub beta cells have also been described as less mature than other beta cells (Johnston et al., 2016). Another distinct subpopulation, the so-called “virgin” beta cells (lacking Ucn3 maturation marker expression), which has been identified as a presumptive beta cell neogenesis niche, is also immature (van der Meulen et al., 2017). Together, these reports suggest that beta cells cannot maintain multiple responsibilities. In other words, a single beta cell cannot successfully secrete insulin in a glucose-dependent manner and replicate simultaneously. Thus, theoretically, the targeted expansion of a proliferating beta cell subpopulation may lead to enrichment in proliferation-prone but physiologically suboptimal subtypes with low levels of insulin synthesis and secretion. Hence, obtaining the right balance between the various types of beta cells appears to be crucial (Nasteska et al., 2019). However, it remains feasible that a population of proliferation-competent beta cells would eventually diversify into beta cell subtypes, under the direction of growth factors *in vitro* or endogenous cues *in vivo*.

Given the subtle nature of beta cell proliferation and difficulties detecting the rare proliferating beta cells, modern transcriptomics is very useful, making it possible to identify replication-associated gene expression patterns, signaling pathways, and finally, mitogens. However, this technology bears certain limitations. Foremost, changes in the gene expression on the level of mRNA do not always translate to the protein level (Chen et al., 2018). Therefore, other high-throughput techniques, such as, for example, Mass Spectrometry (MS) proteomics, Chromatin-immunoprecipitation (ChIP-seq), or Assay for Transposase-Accessible Chromatin (ATAC-seq) has been utilized as well for studies on beta cell proliferation. For example, El Ouaamari et al. (2016) used MS-based proteomics analysis to identify SerpinB1 as a beta cell mitogen. Wang et al. (2016) using single-cell mass cytometry analyzed various pancreatic cells, and not only confirmed (via Ki67 protein labeling) that beta cells replicate the highest during the neonatal period, but also identified several upregulated proteins in proliferating beta cells, such as PDGFRA, pERK1/2, pSTAT3, and pSTAT5. ChIP-seq or ATAC-seq will likely shed light on the epigenomic landscape of the proliferating beta cells leading to identification of new putative mitogens as shown for example in Ackermann et al. (2016) and Sobel et al. (2021). High content screening (HCS) systems allow rapid and reliable testing of large numbers of compounds in an automated manner. The systems must be sensitive enough to detect the delicate changes of labeling markers in the dispersed or intact islets. Successful screening systems of such type have been employed for example by Aamodt et al. (2016), Dirice et al. (2016), and Abdolazimi et al. (2018). Moreover, recently, Yang et al. (2020) managed to engineer a multifunctional screening platform for high-fidelity measurement of proliferation in 3D culture systems of SC-derived beta cells. Findings from transcriptomics and other high-throughput technologies facilitate the formulation of hypotheses based on computational analysis, thereby guiding subsequent experimental research into increasing yields for beta cell derivation *in vitro* and the replenishment of beta cells *in vivo*.

## Risks Associated With Forced Beta Cell Proliferation

Safety is a key issue determining whether any experimental therapy can become clinically applicable. Tumorigenesis is the principal risk associated with the manipulation of beta cell proliferation, as forcing beta cells to proliferate is akin to disrupting the intrinsic anti-oncogenic mechanisms. A high degree of proliferation is the major hallmark of carcinogenesis (Hanahan and Weinberg, 2011). However, in cancer, cell proliferation is characterized with a chronic and uncontrollable manner (Hanahan and Weinberg, 2011; Feitelson et al., 2015). By contrast, physiologically normal proliferation during development and regeneration is tightly regulated by complex signaling networks (Pan, 2007; DeBerardinis et al., 2008; Kim and Jho, 2014). Moreover, cancer cells are also characterized by many well-known features, including genomic instability, modified energy metabolism, the evasion of immune destruction, apoptosis, cellular identity erosion, and metastatic behavior (Hanahan and Weinberg, 2011).

One hallmark of cancer — the distortion of cellular identity — is a matter of particular concern, as several studies have suggested that proliferation provokes a partial loss of beta cell identity, rendering the cells less mature and compromising their ability to secrete insulin (Klochendler et al., 2016; Puri et al., 2018; Xin et al., 2018; Rosado-Olivieri et al., 2020). Controversially, these observations were not confirmed in other studies (Kondegowda et al., 2015; Dirice et al., 2016; Son et al., 2019; Ackeifi et al., 2020b), and some even described an enhancement of the mature phenotype in replicating beta cells (Wang P. et al., 2019; Ackeifi et al., 2020b).

The influence of proliferation on beta cell identity therefore remains a matter of debate and thorough explorations of the effects on cell identity in proliferating beta cells are required, with determination of the extent to which the identity of beta cells can be modified whilst remaining within physiologically normal conditions. The goal of any potential treatment for diabetes is, thus, to stimulate beta cell proliferation without the development of cancer hallmarks. To do so, proliferation must be transient and strictly controlled.

Notably, insulinomas, the beta cell tumors, are rare and benign (Grozinsky-Glasberg et al., 2015), and this may be a positive sign for expansion of the beta cell population *in vivo*. However, the cells surrounding the beta cells may also be affected by mitogen treatment *in vivo*. The neighboring cells may possess significantly lower thresholds for the stimulation of proliferation or may have different levels of cell cycle regulatory proteins. For instance, alpha cells have a higher proliferative capacity and express cell cycle inhibitors at lower levels than beta cells (Gutierrez et al., 2018). Furthermore, pancreatic ducts can transform into malignant cells, giving rise to pancreatic ductal adenocarcinoma (PDAC), one of the most aggressive and lethal human cancers, with a median 5-year survival of 6% (Kamisawa et al., 2016). Importantly, many of the CCRs discussed here have been implicated in PDAC development, including the mutation-mediated expression dysregulation of p16, one of the key driver mutations of PDAC (Kamisawa et al., 2016). Other common CCRs associated with PDAC include CDK6 (Waddell et al., 2015), cyclin D1 (Witkiewicz et al., 2015), cyclin E1 (Bailey et al., 2016), p15 (Witkiewicz et al., 2015), pRb1 (Witkiewicz et al., 2015), and Myc (Witkiewicz et al., 2015; Bailey et al., 2016). It will therefore be essential to prevent the mitogens from acting on non-beta cells.

Conversely, the mitogens currently available have repeatedly raised concern due to their poor specificity (Belgardt and Lammert, 2016; Sabir et al., 2018; Wang P. et al., 2019; Ackeifi et al., 2020b). The primary targets of mitogens (i.e., DYRK1A, TGF-beta signaling effectors) are not expressed specifically by human beta cells (Abbassi et al., 2015; Dirice et al., 2016), and the mitogens themselves have a plethora of additional targets, which remain incompletely elucidated (Ackeifi et al., 2020a). However, modified versions of DYRK1A inhibitors, with improved specificity and efficacy, and lower levels of cytotoxicity, have recently been reported (Kumar et al., 2018; Allegretti et al., 2020).

Hence, due to the risk of PDAC and other cancer type development, as well as the multiple targets of mitogens, the perspective of *in vivo* beta cell expansion for the diabetes treatment remains elusive. Furthermore, the use of beta cell expansion techniques *in vivo* would also require technologies for monitoring changes to the beta cell population *in vivo* that are not currently available (Demine et al., 2020). Given these limitations, a treatment based on beta cell *ex vivo* or *in vitro* expansion followed by transplantation back into the patient’s pancreas may be more feasible and attractive nowadays. Conceivably, the design of such a treatment could combine differentiation or reprogramming strategies, followed by expansion *in vitro*, before the final transplantation of the beta cells back into the patient.

Since mitogens can induce proliferation of alpha cells as well as other endocrine cell populations, the targeted mitogen delivery could be needed for increasing beta mass. Since glucagon-like peptide-1 receptor (GLP1R), that binds GLP1, in human pancreatic islets is expressed only in beta cells (Segerstolpe et al., 2016) and delta cells, the strategy to use GLP-1 as a carrier may be adopted to target/deliver any other small molecule to beta cells. Finan et al. (2012) was able to deliver estrogen to beta cells using the engineered GLP-1 as a peptide carrier. Since then, many examples of GLP-1 analogs for targeting beta cells for diabetes have been engineered (Alavi and Ebrahimi Shahmabadi, 2021). Recently, it was shown that GLP-1–estrogen conjugate treatment increased beta cell number, as compared with those in diabetic mice treated with a vehicle (Sachs et al., 2020). Importantly, GLP-1–estrogen conjugate, but not monoagonists, selectively targets beta cells. Others showed that using a similar GLP1-based system it was possible to deliver antisense oligonucleotides to beta cells (Ämmälä et al., 2018). Researchers were able to silence FOXO1 mRNA that led to a reduction in FOXO1 protein levels in beta cells in a GLP1R-dependent manner both *in vitro* and *in vivo*. Finally, an interesting approach for targeted delivery of harmine to beta cells was described. Based on the observation that beta cells have over a million-fold higher Zn(II) concentration (10–20 mM) compared to alpha cells (1 nM) and other cell types (∼400 pM) (Egefjord et al., 2010; Solomou et al., 2015) researchers reasoned that hydrolytic reactions catalyzed by an excess of Zn(II) (Lemaire et al., 2012; Li, 2014) could be used to deliver drugs to zinc-rich beta cells. Indeed, Yang et al. (2020) designed a zinc-binding prodrug (ZnPD) consisting of three components: an inactive form of harmine, a Zn(II)-binding ligand, and a self-immolative linker. Upon zinc chelation, ZnPD releases their cargo, for instance active harmine. In the 3D-reconstructed culture system increased harmine activity through SC-beta cell proliferation was observed (2.4-fold increase compared to harmine) as well as higher targeting efficiency, and decreased toxicity compared to unmodified harmine. The above-described strategies hold the potential to simultaneously deliver one or more mitogens. In addition, such targeted delivery may enable reduced dosing of the individual components and consequently reduce the risk of unwanted side effects.

## Conclusion

The review describes recent advances and novel directions in research on human beta cell proliferation, focusing on mitogen synergy and the contribution of RNA-seq technologies. It specifically aims to bring the concept of mitogen synergy to the fore and to provide evidence of its promise as a possible route toward a cure for diabetes. Systemic and combinatorial approaches are ubiquitous in both experimental research and modern clinical drug development as complexity of biological systems requires complex solutions. The advances of molecular biology have provided the means to develop such solutions. Transcriptomics technologies are particularly suitable for exploring beta cell proliferation at an unprecedented resolution. Contribution of additional high-throughput tools such as single cell ATAC-seq, perturb-seq or proteomics would aid even more for the elucidation of the subtle mechanisms of beta cell proliferation. It is now necessary to fine-tune the effects of mitogens to master the regulation of beta cell replication and prevent adverse effects, such as carcinogenesis. Therefore, rigorous studies are required to increase the beta-cell specificity of mitogens or beta cell specific mitogen delivery. In addition, the annotation of the signaling events that occur in beta cells after cell cycle reentry is crucial, since the downstream signaling network ‘maps’ of the mitogens could allow the development of mitogen combinations based on the accumulated knowledge. An exciting outlook is the engagement of artificial intelligence (AI) technologies which are already rapidly becoming engrained in the management of diabetes in patients (Li et al., 2020). Prospectively, the mined data about the mitogen effects on beta cell proliferation in combinations and alone could eventually be used for machine learning to build algorithms for the compilation of new combinations. Considerable attention should also be devoted to investigating the peculiar relationships between the processes of maturation and replication in beta cells.

Approaches for controlling beta cell replication, *in vivo* or *in vitro*, have considerable potential for replenishing beta cells in diabetic patients. Perhaps, rather than a single beta cell expansion strategy, a combination of several strategies for beta cell replenishment, including the derivation of beta cells from human PSCs will ultimately make it possible to transfer experimental findings into clinical practice. Given the rapid pace of research on beta cell regeneration, it is reasonable to anticipate such therapies becoming a reality in the foreseeable future.

## Author Contributions

KB and ES reviewed the literature and prepared draft of the manuscript, figures, and tables. MB reviewed the data and wrote the manuscript. All authors contributed to the article and approved the submitted version.

## Conflict of Interest

The authors declare that the research was conducted in the absence of any commercial or financial relationships that could be construed as a potential conflict of interest.

## Publisher’s Note

All claims expressed in this article are solely those of the authors and do not necessarily represent those of their affiliated organizations, or those of the publisher, the editors and the reviewers. Any product that may be evaluated in this article, or claim that may be made by its manufacturer, is not guaranteed or endorsed by the publisher.
